# How acceptable is it for HIV positive African, Caribbean and Black women to provide breast milk/fluid samples for research purposes?

**DOI:** 10.1186/s13104-016-2326-6

**Published:** 2017-01-03

**Authors:** L. Kapiriri, W. Tharao, M. Muchenje, I. M. Khatundi, F. Ongoiba

**Affiliations:** 1 Department of Health, Aging and Society, McMaster University, 1280 Main St. W., Hamilton, ON L8S 4M4 Canada; 2 Women’s Health in Women’s Hands, Carlton Toronto, ON M5B 1J3 Canada; 3 Women’s College Hospital, Toronto, ON Canada; 4 Africans in Partnership Against AIDS, 314 Jarvis Street. Suite 101, Toronto, ON M5B 2C5 Canada

**Keywords:** Afro-Caribbean and Black women, Racial/ethnic groups, Participation in research, Breast milk/fluid samples, Cultural beliefs

## Abstract

**Background:**

The African, Caribbean and Black communities have been found to be reluctant to participate in health research in North America. This is partly attributed to historical experiences as well as their cultural beliefs. Cultural beliefs about the uses of breast milk/fluids could further hinder the participation of African, Caribbean, and Black communities in research involving the collection of breast milk/fluids samples.

**Methods:**

We conducted 17 in-depth interviews and three group interviews (n = 10) with HIV+ African, Caribbean and Black women living in Ontario, Canada to explore their cultural beliefs about breast milk/fluids and their acceptance of participating in research that involves the provision of breast fluid samples.

**Study design:**

Qualitative study involving in-depth interviews.

**Results:**

Our respondents believed that breast milk/fluids should be used for infant feeding and for curative purposes for a variety of children’s health ailments as well as ailments experienced by other family members. The cultural belief that breast milk/fluids could be used to bewitch the baby and mother and the perception that it is intrusive (equating breast milk/fluids research to DNA testing), could prevent African, Caribbean and Black women from participating in research involving the collection of breast milk/fluids. Despite these fears, some respondents expressed that they would participate if the research results would benefit them directly, for example, by finding a cure for HIV, enabling HIV+ mothers to breastfeed, or contributing to developing new drugs or vaccines for HIV. Women’s recommendations to facilitate successful recruitment included giving incentives to participants, and employing a recruiter who was trustworthy, informed, and culturally sensitive.

**Conclusion:**

Cultural beliefs could present barriers to recruitment and participation of Africa, Caribbean and Black communities in health research involving breast milk/fluid samples. Successful recruitment for future studies would necessitate researchers to be culturally aware of the beliefs held by African, Caribbean and Black women, to build trust, and use an appropriate recruiter. While the findings relate to breast milk/fluids, the suggested recommendations for facilitating recruitment of research participants from these communities may be useful to consider when recruiting ethnically and culturally similar participants for research involving biological samples.

**Electronic supplementary material:**

The online version of this article (doi:10.1186/s13104-016-2326-6) contains supplementary material, which is available to authorized users.

## Background

There is evidence that racial and ethnic minority groups are sometimes reluctant to be involved in health research. This is partly attributed to historical experiences as well as their cultural beliefs [1, 2]. For example, cultural beliefs among the African, Caribbean and Black (ACB) populations about the uses of biological samples, specifically breast milk/fluids, could hinder recruitment of participants in this kind of research. While blood, urine, saliva and organ tissue samples are collected from participants for research purposes, [3] we hypothesize that ethnicity and cultural beliefs may influence the acceptability of ACB women to provide their breast milk/fluids for research purposes.

The collection of biological samples from people living with HIV has, and continues to contribute to our understanding of the disease and finding a cure. Most of the studies to date have focused on blood, urine and tissue samples. However, since only 15% of infants who are breastfed by HIV-positive mothers become infected with HIV [4], there is now increasing interest in studying breast milk/fluids. Studies suggest that the majority of babies who do not become infected despite repeated, high and consistent exposure to the virus in the breast milk could be protected by immune factors in the milk. Human milk contains a wide variety of relatively uncharacterized innate immune factors and some (e.g. lactoferrin) have been shown to have anti-HIV activity. Characterizing the levels of specific innate factors in human milk or breast milk/fluids may potentially inform HIV vaccine development. Determining whether innate factor levels in human milk correlate with postnatal HIV transmission or account for the protective benefits of exclusive breast feeding would necessitate collecting and studying breast fluid samples from multi-ethnic HIV-positive and negative mothers. The presence of people from different ethnicities within Canada would provide researchers the opportunity to explore how the innate factors within the breast milk/fluids vary (if at all) across different ethnic groups. Understanding these variations could potentially inform the development of a vaccine that would be effective across different ethnicities. However, there might be cultural barriers that might discourage ethnic women’s participation [5, 6].

Findings from studies that have explored the influence of ethnicity and culture on enrollment in any kind of health research are somewhat conflicting. ACB communities in the USA are less likely to participate in health research and clinical trials compared to other ethnicities due to historical experiences of racism and discrimination and unethical research practices [7, 8]. Shavers-Hornady et al. [9] found that distrust, poor access to care, lack of active recruitment, alienation of minority doctors, lack of knowledge and cultural beliefs influence the involvement of African American participants in health research. Sheikh et al. [10], who explored the willingness of South Asians to participate in an Asthma study, found researcher attitude, stereotyping, logistical challenges and poor understanding of the research to be important factors. In addition, Hughes et al. [11] found that cultural beliefs hindered African American women from participating in genetic risk assessment studies, while Corbie-Smith et al. [12] reported mistrust of the medical communities as a barrier for these populations to participate. Contrary to these studies, however, Filippi et al. [13] and Wendler et al. [14] found no statistical significant differences in willingness to participate in health research between different ethnic groups.

While this literature is informative, none of the studies focus on research on breast milk/fluids among HIV+ ACB women [15–20]. We chose to recruit ACB women because according to the literature, they are more likely to breastfeed their children and hence, in cases where they cannot breastfeed (e.g. when infected with HIV), they would stand to benefit most if there was a breakthrough in developing a viable vaccine. A study that explores the acceptability of HIV+ ACB women to provide breast milk/fluid samples would inform researchers who might wish to collect breast milk/fluids, or other biological samples from these populations.

### Study objectives

1. To describe the cultural beliefs about the uses of breast milk/fluids, among HIV+ African, Caribbean and Black women (ACB) in Hamilton and Toronto Ontario, Canada.
2. To establish the acceptability of using breast milk/fluids for research purposes.
3. To determine the culturally acceptable approaches to collecting breast milk/fluids and other biological samples from these populations.

#### Ethics

This study was reviewed and approved by the McMaster University Research Ethics Board. The study participants signed a written consent and received compensation of CAD$50. While confidentiality was ensured during the interviews and after the data were collected, it is not always possible to ensure that everyone participating in a group interview observes confidentiality. This was explained to the participants, and they were encouraged not discuss the proceedings of the discussion outside the room.

## Methods

This was a qualitative study involving 17 interviews and three group interviews (n = 10). The group discussions were used to validate the findings from the interviews, and explore some of the related sensitive topics e.g. criminalization, that arose from the interviews. Both the individual and group interviews were conducted by a trained research assistant (RA) and the Principle Investigator (LK) both of whom were female and of ACB background. There was no relationship between the researchers and the participants. This paper is part of a larger project that was informed by a phenomenology theoretical framework. The larger project explored the lived experiences of HIV+ ACB women making infant feeding choices. The themes of women’s acceptance of using breast milk/fluids for research emerged during the interviews and data analysis [21].

### Setting

The study was conducted in Hamilton and Toronto, Ontario, Canada. Individual and group interviews were conducted in private locations identified by respondents.

### Participant recruitment

We employed mixed strategies to recruit participants for both the individual and group interviews. We recruited adult ACB women (>18 years), who had had a child and were HIV positive. Initial respondents contacted the researchers by telephone in response to posters posted at HIV support organizations. We also requested the respondents we interviewed to pass on the recruitment information to other women who met the criteria and would be interested in participating.

### Data collection

Since all the respondents, with the exception of one, were fluent in English, we conducted the interviews in English. A pre-tested interview guide was used where participants were asked about: (1) their cultural beliefs about breast milk/fluids and, (2) Specifically, how acceptable it would be for them (and their communities) to provide breast milk/fluids for research purposes. While similar themes were discussed further in the group interviews, these interviews focused mainly on following up on themes that had seemed sensitive during the individual interviews. During the interviews, the interviewer/facilitator maintained an open stance and followed up any new information that emerged from the responses. The interviews were audio-recorded, with permission from the respondents. The interviewer took detailed notes for one interview where the respondent declined the recording. The interviewer also kept field notes and a reflexive diary after each interview [22]. These were integrated into the data analysis.

Each interview lasted between 45 and 60 min; while the group interviews lasted between 90 and 120 min. The audio-recordings were transcribed verbatim. Both the PI and the RA read through the transcripts during data collection to identify any emerging themes that were followed up in the subsequent interviews. Sampling ended when there were no new themes emerging from subsequent interviews [22].

### Data analysis

Interview transcripts were analysed using a thematic approach with the interview as the unit of analysis. Data were free coded, to identify key concepts that emerged from the interview transcript. This process involved the PI and research assistant manually coding the same interview and meeting to discuss and compare the concepts identified and code names applied; an agreed upon code list was generated. The agreed upon list of code names was used to code the rest of the interviews using NVivo10. Once all the interviews and the group interview transcripts were coded, at an abstract level, related codes were grouped together under categories. Similar categories were then grouped together to form themes [23]. To ensure validity, our preliminary interpretation of the data were presented and discussed with a group of HIV+ ACB women who had participated in the study (also called member check).

## Results

All the respondents and group participants, with the exception of three, were immigrants from either Africa or the Caribbean. Their ages ranged from 30–60 years (all with the exception of two, were below the age of 40, and had children within 10 years prior to the study).

The results section is organized in the order of the study objectives. Illustrative quotes from the interviews are provided, where appropriate. Synonyms used include: G: Group Interview (I, II, III); R: Individual interview (numbers were arbitrarily assigned).

### 1. Uses of breast milk/fluids: cultural perspectives

All interviewees, with the exception of one, had children whom they had breast fed; and so were conversant with the cultural beliefs about the uses of breast milk/fluids. The most common and acceptable use of breast milk/fluids was infant feeding. Therapeutic uses of breast milk/fluids were also described in which breast milk/fluids were used for the treatment of baby’s ear, eye or for the treatment of genital infections, and nasal congestion. In a few interviews, breast milk was said to provide a cure for alcoholism. Combining breast milk with alcohol was described as causing severe nausea, which then prevented an alcoholic from ever desiring alcohol drink again. In this context, the importance of limiting breast milk to therapeutic use by the core (nuclear) family was emphasized, based on the perception that use outside the family could cause harm to the mother or the infant. As illustrated by the following quote, using it outside the family increased the potential for malevolent use.

*“…If you use it outside the family… we believe that someone can do bad things by witchcraft…because witches they use different things …so you have to use it in the family…you can’t use it outside the family…” (G II)*

#### Beliefs about using breast milk/fluids for research

In most interviews, the respondents’ acceptability of providing breast fluid samples for research purposes was offset by reservations tied to cultural beliefs about breast milk. Moreover, their acceptability of participating was related to an understanding of the potential usefulness of research.

*“…We should help the researchers….”*

Most of the respondents said that they, and their “communities”, would provide samples since they have gained understanding of research, its purposes, and potential benefits. They also felt well protected by the legal system in Canada. With regards to understanding research, the respondents reported that since coming to Canada, the exposure and their participation in many research studies has provided them with a better understanding of research generally, and thought it relevant for them to support researchers.

*“…I remember when HIV started…many people did not want (to hear) about research…but now more people are open to research coz they give information on HIV. People now are (more) familiar with research…” (R3)*

“…*it is very important to help the people who do the research to know….maybe if a mother has HIV….that’s how they know…” (GI)*

The second reason for their acceptability was the potential benefits of the research. The respondents recognized the fact that most of the progress made in discovering the HIV virus and treatment has been based on research. Respondents reported that the people living with HIV, are eager to have a cure; *“…to make HIV history…”* Specifically, they would be motivated to participate if the purpose of the research is to develop an intervention that would enable the women to breast feed their children despite their HIV status.

*“… If it’s not the research they would not have known…If the medication they give people living with HIV to reduce the virus from developing in the blood (is effective)…if we hadn’t taken the blood tests…they would not know, research is very important…” (R4)*

The third explanation was quite interesting and clearly demonstrated that the respondents have been sensitized and are aware of their rights as study participants. They said they would be more inclined to participate in research within Canada as compared to their home countries, since in the Canadian context, it is unlikely that their rights, as study participants, would be abused.

However, a few respondents had reservations. We discuss the few variant responses in detail.

- “…What if someone bewitches me?

A sample of women expressed fear about providing breast fluid samples. This was grounded in their cultural beliefs that someone could take the samples under the pre-text of research and use it to bewitch and cause harm to the baby and/or the mother. Respondents thought that the chances of this happening would be greater if they were in their home countries where they are known. In such cases, the only people they would trust would be the health workers, who they assumed, would not know them personally. Within the Canadian context (or when away from “home”), the fear of providing breast milk/fluids for research purposes still existed, but was downplayed due to the anonymity that being an immigrant gave to the potential participants.

*“…I think especially back home people believe in so many things…in Africa you know… witchcraft…so if somebody comes and asks you for your breast milk…someone will say…mmh he wants to go and do something with it to bewitch me. But if I’m here in Canada and somebody comes and says I am doing a study and I am looking for women to give me some of their breast liquid..and I know it’s a study…there are many studies here… for example research to get medication for cancer or for HIV…so I don’t refuse if I am in this country (Canada). So it depends…for me now if I am back home and somebody come and ask me as long as the person is a medical nurse then I’ll know she is really doing it for research..I don’t see a reason why I will refuse…but my community will not think that way… they will refuse because they have this mind that you will go and use it for witchcraft…” (RI)*

One respondent reported that some women may fear providing samples because they are worried about being “exposed” to the public. According to this respondent, these people regard breast milk/fluids as “private and personal”; hence, giving breast milk/fluids samples could be compared to giving their DNA. So providing breast milk/fluids for research purposes carries the possibility of exposing participants to the public, which was invasive and undesirable.

*“…Ooh I don’t think anybody will go handing over samples of their breast milk/fluids….yah I don’t think anybody would agree to that….yah…it would be kind of invasive I think it is kind like giving out your DNA…yah…” (R2)*

The other fears expressed with regards to participating in such a study related to the recruitment strategies and if the participants’ HIV status would unknowingly be revealed. Some participants thought that the possibility of people finding out the participants’ HIV status would scare potential participants. For example, a study advertisement that specifically said they are recruiting HIV positive women—had the potential to reveal the participants’ HIV status, especially if conducted in an “unsafe” environment. Another source of fear related to individuals discovering their HIV status through their participation in the study. There was a concern that some people do not want to know their HIV status; therefore a study on breast milk/fluids might reveal their HIV status to them, with the attending consequences, as expressed in this quote:

*“….. maybe someone in the community don’t want to know about their HIV status because they feel it will make them unhappy, stressed, traumatized or depression so they prefer not to know…if I know nothing, I can live long with my virus. They don’t want(to know) because it will make them scared. It will make them feel stigmatized or discriminated…” (R4)*

Furthermore, one respondent reported the possibility of peer influence whereby if one person refused to participate in such a study (due to misunderstanding of its purposes), they could easily influence others within their communities, misinform them and advise them not to participate.

*“…They will not accept…I just know they will not accept…I don’t know if it is not understanding…or it is because we are still behind…even someone I know will tell others…that if you participate…you will get breast cancer…don’t participate…they will just look for reasons…" (R6)*

We followed up with the respondents who said it was acceptable to participate in a study involving providing breast fluid samples by asking them to provide their advice with regards to making such a study culturally appropriate and feasible.

### 2. Practical advice for collecting breast milk/fluids: the women speak out

The respondents provided practical advice with regards to obtaining biological samples from their communities. Their advice can be summarised under 3 categories: (i) *The need to foster understanding about the benefits of the research*; (ii) *Recruiting and motivating participants* (iii) *Practicalities of sample collection*. We explain these in detail below:

#### i. The need to foster understanding about the benefits of the research

Given the potential for misunderstanding, the participants recommended that community education about the study should precede the recruitment of individual participants. This education would prevent individuals from misinforming others and discouraging them from participating. Such an approach was perceived to be the best way for researchers to enter into these communities when conducting a study which may be culturally sensitive. The participants explained that lack of understanding creates suspicion that deters people from participating.

*“…before you go and ask for each and every person for their milk you have to call them for a meeting and explain the reason why you are doing the study (R7)…”*

The respondents emphasized the need to highlight the potential benefits of the study. Examples of potential benefits that would motivate the study participants included: finding a cure for HIV, enabling HIV+ mothers to breastfeed, and contributing to developing new drugs or vaccines for HIV. These ideas are evident from the following quotes:

*“…they (the communities) are eager for medication to be found and HIV to become history…because many have perished because of HIV…” (R5)*

*“…if you tell them you are doing a research to find out if it is possible for HIV (*+ *mothers) to breast feed, everybody will be willing to take part because some are HIV* + *and they don’t have money and they want to breast feed so I don’t think they will have a problem with that…” (R6)*

After establishing the motivators for participating in a breast fluid study, participants also proposed strategies for recruiting participants.

#### ii. Recruiting and motivating participants

Respondents emphasized the need for women to be able to trust the person who is recruiting them into the study. The attributes of such a person included: a female, someone who is known and ‘respected’ in these communities (a doctor or a health worker who already works with the women). The preference for the health worker partly related to their anonymity and the already existing relationship.

The recruiter should also be ethnically “relevant” to the communities. Participants would prefer someone who understood their culture and “context”. Some suggested having a recruiter from that given “community”—since “…it is easier to trust such a person…” This person should know (and “understand”) not only the research topics, but also the social and cultural “worlds” of the HIV+ ACB women participants.

*“…yah like HIV (people) they know their own people…you use that person…and people who work in the field…because they have a lot of trust…” (R3)*

*“… definitely it should be a woman who can explain…what she wants the samples for. She should understand them (the participants)…the group that she’s going to deal with because there are different classes; low class, middle class, high class. She should know the group that she is targeting…and have the right words…” (R8)*

However, that idea was not acceptable to all respondents since a few thought using someone from within the communities would threaten their anonymity.

Recognizing the opportunity costs for participating in the study, participants recommended that the women should be compensated for their participation, for the time they spend and the fact that the researcher “benefits” from the research.

#### iii. Practicalities of sample collection

Respondents were asked about practical tips they would give to someone who would like to succeed in conducting a study involving collecting breast milk/fluids. They emphasized the importance of the location where the samples would be collected and the person responsible for collecting the samples.

With regards to the locations, all the respondents agreed that a “good” community health centre or hospital would be most appropriate. These were places that are familiar with and routinely provide services for the HIV+ women. The timing of collecting the samples was also important. The respondents advised that the samples should be collected around the time the woman starts to produce colostrum and when she woman needs to come to the health unit for routine check up. Using the usual places and time would also reduce the potential for stigmatization and the inconvenience for the women.

*“…you have also to choose a hospital or community health centre. Then they will know that this is for research. The place is very important, a hospital and community centres…” (R3)*

*“….are they going to collect it(breast milk/fluids) in the hospital where the mother is…that’s different but once she’s gone home and telling them to come back for(collecting the sample)….it doesn’t make sense…” (GI 6)*

Respondents also emphasized the importance of having the “right” person collecting and/or supervising the collecting of the samples. According to participants, this person should have characteristics similar to the recruiter described above.

## Discussion

ACB communities have been found to be less willing to participate in health research. This study contributes to this literature by examining the cultural beliefs of HIV+ ACB women about the acceptability of providing breast milk/fluids for research. To the best of our knowledge, this is the first study that addresses this issue within Canadian and similar contexts.

The finding that some of the respondents would find it unacceptable to provide breast milk/fluid samples was closely connected to their cultural beliefs that breast milk/fluids should be used to benefit the infant or family members. The belief that breast milk/fluids can be used as a medium for witchcraft could be a potential barrier that researchers might need to address to facilitate ACB women’s involvement in such research. However, within contexts where anonymity is ensured, this belief may have less impact. In contexts where anonymity cannot be assured, trust needs to be established.

Cultural beliefs are not the only potential barriers to HIV+ ACB women’s acceptability of providing breast milk/fluid samples. In high-income countries where HIV+ women are not allowed to breastfeed their babies, women may experience psychological and emotional distress about providing breast fluid samples [24]. The acceptance of these women, in spite of this, to participate could be interpreted in several ways; first women might view the potential benefits of the study (e.g. enabling them to breast feed) to outweigh the potential distress. Additional explanations include women’s awareness of the role that research plays in facilitating the discovery of drugs to treat HIV. The other explanation could be fear. Since the participants were immigrant women, there might be the belief/expectation that they have to participate in research that is hosted in their host country to be considered “good citizens”, or else face consequences [25]. The third explanation could be the possibility of getting money for participating in research. These explanations could be applicable to similar studies even if they may not involve HIV+ women, and raise ethical concerns that are beyond the scope of this paper.

It was surprising that our respondents did not identify historical experiences with research as a reason for their reluctance to participate in research since the literature about research among ACB highlights this as a barrier [2]. It is possible that most of the respondents were immigrants who may not be aware of the negative history of research on ACB populations in North America. Conversely, as the participants reported, they trusted the Canadian system to protect their rights, which is a positive finding.

The practical recommendations that the respondents made to facilitate a culturally sensitive study were realistic and congruent with the current literature—trust between the researcher and the communities involved is very critical to these populations [26]. Successful recruitment of participants from these communities hinges on whether or not they trust the researcher (and the person recruiting the participants). Confidentiality, though relevant in all studies involving human participants, may be more critical to HIV+ ACB populations since revealing their HIV status may result in partner violence, stigmatization and isolation [27].

The respondents’ recommendations for the recruiters’ attributes should be considered when soliciting the participation of these communities. However, the examples of such a person provided, i.e. someone who is sensitive to their cultural and social contexts, and the involvement of health workers, is somewhat contradictory and raises concerns. There was disagreement among women as to whether the culturally similar person should come from their communities—and be known to them; or whether this person should be sensitive to their culture and understanding but not necessarily be from their communities. The dilemma here was balancing the benefits of being culturally understood and remaining anonymous. These findings emphasize the need to observe cultural sensitivity and confidentiality in research. Where peers are used, this should be emphasized. However, for people who may not be comfortable disclosing information they consider to be sensitive such as their HIV status to the immediate peers, it may be useful to consider using peers from other locations; this way, participants can maintain their anonymity.

While discussing culture, the difference between culture and ethnicity should be noted. Just because someone is black does not mean that they hold the same cultural beliefs and practices as all the other black people [28]. As well, it is faulty to assume that just because someone is of a certain ethnicity they understand the cultural beliefs and values held by the group they are researching. In addition to training in the basic principles of research, peer researchers need to be aware of the cultural beliefs that are relevant to their study populations.

## Conclusions

This paper identifies the cultural beliefs about the use of breast milk/fluids for research. While participants showed interest in participating in such a study, the cultural beliefs that the mother and baby could potentially be harmed through the fluids and fear of being “exposed” could hinder their participation in such studies. Understanding these cultural beliefs (and the historical context of research in these populations) and using them as a basis to foster common understanding between the communities, researchers and health workers would be useful in reaching these communities.

To the best of our knowledge, this is the first study that explores cultural beliefs with regards to research that uses of breast milk/fluids within the Canadian context. While the focus was breast milk/fluids; the findings of the study shed light on cultural beliefs that may be applicable to research involving collecting other bodily samples such as blood, and body tissue. The practical recommendations for collecting bodily samples, for instance, having a culturally sensitive, knowledgeable and trusted person at the frontline, the need for educating communities about the research, the need for incentives to motivate participants, and the importance of ensuring confidentiality, are useful to any researcher who might want to collect biological samples at the community level. While the current literature is full of the need to have culturally aware health care providers, we argue that cultural sensitivity is equally relevant in the context of health research. Researchers and their research assistants need to have knowledge of the cultural values (relevant to their research topic) of the populations they wish to investigate, before conducting their research.

This is an exploratory study that points to important findings with regards to the participation of one minority group in research. There is need for similar research with representative samples of respondents.

## Limitations

This was a qualitative study, involving a small sample of respondents. While the results may not be generalizable, they provide insight to a specific issue and could be applicable to similar populations. The study findings should also be interpreted with caution since we interviewed people who contacted us and expressed their interest in participating in our study. The sample might represent a biased population that regularly participates and is very knowledgeable about research, and hence, may not be representative. However, the use of posters is one of the standard ways of recruiting study participants. Furthermore, our initial results were presented and validated by a wider group of ACB women living with HIV, some of whom did not participate in the study.

## Additional file

**Additional file 1.** Interview guide.

## Abbreviations

HIV: human immune deficiency virus
ACB: Afro-Caribbean and Black
