# Supplementary material for: How acceptable is it for HIV positive African, Caribbean and Black women to provide breast milk/fluid samples for research purposes?
Source: BMC Res Notes. 2017 Jan 3;10:7. doi: 10.1186/s13104-016-2326-6 (PMC5217306; doi:10.1186/s13104-016-2326-6)
Supplement: Supplementary file 1 — Additional file 1. Interview guide. [file 13104_2016_2326_MOESM1_ESM.doc]

**Provisional Interview Guide**

**Psycho- Social, Cultural and Ethical aspects of infant feeding choices for mothers in the era of HIV: Community Based Capacity Building Research Project.**

Thank you for agreeing to participate in this study. Our discussion will begin with a few general questions about yourself.

1. How old are you?
2. Which country are you originally from?
3. How long have you lived in Canada?

*We will now talk about you beliefs and practices with regards to breast fluids and infant feeding*

1. In your culture, what are some of the things that people do when a baby is just born? (Probe for any special feeding ceremonies/ mouth washing/ sweetening)
2. In your culture, what do people think about the proper way to feed a new born baby?
3. What are people usually told in the hospitals in your home country—with regards to feeding new born babies?
4. What are the similarities/ differences between your experiences with infant feeding in your country of origin and here in Canada?
5. In your culture, what do people believe about the uses of breast fluids?
6. In your culture, what are people’s beliefs about providing breast fluids for research purposes? (Probe for how acceptable it is and explanations behind the beliefs)
7. What advice would you give a researcher who might want to conduct such a study, with regards to what you believe in your culture?(Probe for in Canada and in the home countries; facilitators and barriers)
